# Performance measures for substance use disorders – what research is needed?

**DOI:** 10.1186/1940-0640-7-18

**Published:** 2012-09-11

**Authors:** Deborah W Garnick, Constance M Horgan, Andrea Acevedo, Frank McCorry, Constance Weisner

**Affiliations:** 1 Institute for Behavioral Health, The Heller School for Social Policy and Management, Brandeis University, Waltham, MA, USA; 2 Chair, Washington Circle and FAM Consulting, Yorktown Heights, NY, USA; 3 Division of Research, Northern California Kaiser Permanente, University of California, San Francisco, CA, USA

**Keywords:** Performance measures, Research agenda, Process, Outcome, Structure, Electronic health records

## Abstract

In 2010, the Washington Circle convened a meeting, supported by the National Institute on Drug Abuse (NIDA) and the Substance Abuse and Mental Health Services Administration (SAMHSA), for a multidisciplinary group of experts to focus on the research gaps in performance measures for substance use disorders. This article presents recommendations in three areas: development of new performance measures; methodological and other considerations in using performance measures; and implementation research focused on using performance measures for accountability and quality improvement.

## Introduction

Two decades ago, the Institute of Medicine defined quality as “the degree to which health care services for individuals and populations increase the likelihood of desired health outcomes and are consistent with current professional knowledge” [1]. Today, performance measures, the metrics used to measure the quality of healthcare, play increasingly important roles in all aspects of healthcare.

In particular, the increased focus on performance measures that address the prevention and treatment of substance use disorders has been driven by new legislative initiatives. The 2010 Patient Protection and Affordable Care Act has the potential to open up treatment for substance use disorders to more Americans. The 2008 Mental Health Parity and Addiction Equity Act expands benefits for mental and substance use disorders. The Health Information Technology for Economic and Clinical Health Act (HITECH Act) encourages the promulgation of electronic health records and incentives for performance measurement. In addition, widespread payment reforms, including incentive–based approaches, are leading to an ever stronger focus on the accountability of clinicians and treatment programs for providing high quality services.

Given the high stakes uses of performance measures, it is crucially important that performance measures focused on substance use disorders be further developed, be critically examined, and be implemented in the context of a changing health care system [2]. Therefore, in March 2010, the Washington Circle convened a meeting of payers, consumers, providers, policy makers and researchers to discuss the state of performance measures and to develop a research agenda to address current deficits. The Washington Circle is a group of national experts on substance abuse policy, research, and performance measurement that seeks to improve the quality and effectiveness of prevention and treatment services through the use of performance measures [3]. This meeting was supported by the Substance Abuse and Mental Health Services Administration (SAMHSA) and the National Institute on Drug Abuse (NIDA).

Specifically, the participants’ charge was to: review the current status of performance measures focused on substance use disorders; identify gaps in currently available measures and opportunities for research, in light of our current understanding of addictive disease and recovery; and develop a research agenda. Rather than set out a concise list of research priorities, the participants focused on developing a range of suggestions. In reporting on this meeting, our goal is to stimulate new research on performance measurement by drawing connections to and making suggestions about areas that need focused attention.

In this meeting report, we briefly review the context for developing a research agenda for performance measures for substance use disorders and present the recommendations. This report builds on a foundation of earlier work, including the Institute of Medicine Report: Improving the Quality of Health Care for Mental and Substance-Use Conditions [4], a background paper commissioned for that report [5], and a recent article on priorities for policy research [6].

### Context

Timothy Condon (National Institute on Drug Abuse) emphasized that performance measurement needs to be considered in the context of developments in our understanding of substance use disorders, of newly emerging standards of care, and of behavioral health more broadly, as well as in the context of general medical care. Increasingly, common thinking has shifted from considering substance misuse to be a human failing to an understanding that addiction is a treatable disease. Clinical research has revealed the role of genetics in vulnerability to addiction, changes in brain functioning that make voluntary behavior different after addiction than in the absence of addiction, and the role of social/cultural environment in addiction. Thus, the most effective treatment strategies will attend to all aspects of addiction, including biology, behavior and social context. It is key to consider these aspects of the nature of addiction in developing a research agenda on performance measurement [5]. There is an emerging consensus that addiction is often a chronic condition akin to other chronic conditions in the medical sphere with similar rates of non-adherence to treatment and relapse.

Mady Chalk (Treatment Research Institute) pointed out that there is a well-studied repertoire of interventions to treat addiction or to change unhealthy patterns of use which has provided additional impetus to the development of measures. Although there are evidence-based approaches to addiction treatment, their implementation remains challenging because of limitations involving organizational readiness, resources, and leadership [7].

Describing the landscape of performance measurement, Constance Horgan (Brandeis University) emphasized that a decade ago, only a handful of organizations focused on performance measurement, while today a plethora of groups are developing, testing, endorsing, using, or selling measures in the general medical sector. Several leading organizations include a specific focus on performance measures for substance use disorders in their current initiatives including the National Quality Forum (NQF), the National Committee on Quality Assurance (NCQA), and the American Medical Association (AMA). Many other groups are incorporating performance measures for substance use disorders into their broad-based measure development initiatives. One promising measurement approach is to consider individuals’ multiple interactions with the health care system through composite measures which allow for a more integrated assessment of performance that can include behavioral as well as general medical conditions.

### Research recommendations

The meeting participants were asked to focus their recommendations in three areas: development of new performance measures; methodological and other considerations in using performance measures; and implementation research focused on using performance measures for accountability and quality improvement. During the meeting, the participants did not prioritize among their many research recommendations, although there was general consensus that work is needed in all three broad areas. In this section, we review these recommendations and outline their rationales.

#### Development of new performance measures

Developing new performance measures is challenging because they need to meet several criteria, including importance, scientific soundness and feasibility [5,8]. Endorsement by the National Quality Forum (NQF) is required for measures to be used in Federal programs, and the endorsement process imposes a high level of rigor with respect to these standards of specification and testing. The meeting participants recommended development across a balance of types of measures - structural, process, and outcome measures. The group also recommended consideration of composite measures that aggregate multiple domains and stressed the need to establish associations between structural or process measures and outcomes.

#### Development of structural measures

Structural measures are features of a healthcare organization or health system that are focused on its capacity to provide health care. They are often used in contracts between health plans and managed behavioral healthcare organizations, and in accreditation of facilities. For example, in the NCQA accreditation of managed behavioral healthcare organizations, structural measures are included to determine whether there are practitioners located throughout the service area and whether there are sufficient numbers of practitioners [9,10]. Promising areas for development of new structural measures should build on current research on best management practices in the following areas:

• *Inclusion of medications to treat addictive disorders on a health plan’s formulary.* For example, this includes naltrexone, disulfiram and acamprosate for alcohol abuse and dependence, and buprenorphine and methadone for opioid dependence.

• *Rate of collecting and reporting data on clients’ perceptions of care using standardized instruments* (e.g., the ECHO or Modular survey). Surveying clients is consistent with the national priority for patient-centered care.

• *Measures of management practices.* This may include whether treatment organizations have business practices in place that are associated with better treatment processes, such as management practices that have been found to be associated with shorter wait times from first contact to treatment admission [11].

• *Measures of connections between organizational providers across the continuum of treatment services.* This includes a range of efforts that support care coordination including communication, networks, or contract elements between providers of residential and of follow-up outpatient services.

#### Development of process measures

These measures are used to assess a health care service provided to, or on behalf of patients. Often they are used to assess adherence to recommendations for clinical practice based on evidence or consensus. For example, measures of initiation and engagement developed by the Washington Circle [12-14] are already being used extensively by, among others, the National Committee for Quality Assurance [15], the Department of Veterans Affairs (VA) [16,17] and several states [14]. Related measures for access to and retention in treatment have been developed by the Network for the Improvement of Addiction Treatment (NIATx) [18-21]. Clinical performance measures for physicians were developed by the Physician Consortium for Performance Improvement (PCPI), convened by the American Medical Association; these include counseling regarding psychosocial and pharmacologic treatment options for alcohol and opioid dependence, and screening and brief counseling for unhealthy alcohol use [22]. Recently, the Washington Circle released specifications for two new measures on MAT, focused on overall use of appropriate medications for substance abuse disorders and on the timely initiation of medications in newly treated individuals [23,24], although development of additional measures focused on adherence rates and clinical follow-up after initiation of medications is still needed.

While recognizing the advances made in developing process measures, the meeting participants also noted some gaps and the need to develop additional measures, consistent with findings from clinical or services research:

• *Measures to monitor screening and brief intervention services for unhealthy alcohol use.* These measures might take advantage of recently adopted procedure codes [25].

• *Maintenance of treatment effects.* These include recovery support and retention, going beyond counting units and timing of services to also focus on treatment intensity and quality of engagement and the therapeutic relationship.

• *Measures tailored for specific groups of clients.* Based on results of research showing which treatment approaches are most effective for specific groups, measures might be tailored specifically for women, individuals released from incarceration or adolescents.

As Alexander Harris reported (Palo Alto Veterans Administration (VA) Healthcare System), the literature assessing the relationship between process or structural performance measures and outcomes has not received sufficient attention. In the past few years, research has begun to be published on the association of some process measures and outcomes, although the findings are mixed. Clients with a new outpatient treatment episode who engaged in treatment were less likely to be arrested or incarcerated the following year [26,27]. Adolescents in residential treatment achieving continuity of care after treatment were significantly more likely to be abstinent at 3-months post-discharge [28]. VA patients who met engagement criteria had significantly greater reductions in addiction severity than those who did not. However, although statistically significant, those reductions were found to be clinically modest [16].

Further studies are crucial to better understand the nuances of associations between improvements in structural or process performance measures and improvements in clients’ outcomes as well as lower costs or better efficiency in the treatment system. These studies should be focused both on existing performance measures and on new measures as they are developed. Indeed, testing the association of alternate specifications of structural or process measures can be built into routine measure development [29]. Moreover, it is critical to assess if any association varies by client group (e.g., gender, race/ethnicity, or age) or treatment setting.

#### Development of outcome measures

These measures, focusing on health states resulting from health care practices and interventions, generally reflect the cumulative impact of multiple processes of care [30]. For substance use disorders, commonly used outcome measures go beyond the measures of clinical outcomes or functioning commonly used for medical conditions to include non-clinical outcomes such as: stable and supportive housing [31-34]; employment (for adults), educational involvement (for adolescents); and decreased criminal justice involvement. In addition, it is recognized that a broad group of service systems extending beyond the medical sector provide a coordinated menu of services and supports to maximize outcomes, e.g. recovery support, family, housing/homeless, child welfare, education, and criminal justice [35].

Given this context, additional development of performance measures focused on outcomes is needed in the following area:

• *Measures that consider addiction as a chronic condition*[36]*and explore the emerging construct of recovery as it becomes more deconstructed and operationalized*[37]*.* Initial efforts are needed to prioritize which new measures are needed, such as well-being, severity of alcohol or substance use, days of use, functionality in work and home life, and health status. The next step is translating these concepts into measure specifications.

#### Development of composite measures

These measures go beyond single structural, process, or outcome measures. They combine multiple measures to give a broader picture of performance, but they may be complex to interpret and next steps in quality improvement may not be clear. For example, while there is some work on performance measures for co-occurring substance use disorders and mental health problems [38], little research has been focused on performance measures for treatment of clients who have co-occurring substance use disorders and chronic medical conditions. Participants also recommended that new composite measure development should also be considered in the following areas:

• *Composite process measures that aggregate current substance abuse treatment process measures for a specific client.* For example, a composite could include whether the client become engaged in treatment and was offered medication assisted treatment.

• *Composite process measures that incorporate multiple components of the process of care for substance use disorders.* This approach would expand beyond number and timing of treatment services to also include client perception of the quality of visits and client behavior indicative of engagement in the treatment process such as web discourse with clinicians [39].

• *Composite outcome measures at the client level that aggregate multiple outcomes.* This outcome-focused composite might combine abstinence, housing and employment.

• *Composite structural measures focused on whether an organization has a set of structural elements.* Among structural measures to consider are organizational capacity; leadership/management; clinical and administrative supervision functions; documented treatment philosophies; workforce issues/characteristics such as inclusion of case managers, staff training/credentials, staff productivity and performance; financial practices; and organizational culture.

### Methodological and other considerations in using performance measures

In addition to developing performance measures, additional research is needed to critically examine performance measures in three areas: design issues and their impact on measure development and use; new sources of information, particularly electronic health records; and integration of performance measures with settings outside the specialty substance abuse treatment and with performance measures for medical conditions.

#### Exploration of design issues

Both new measure development and research that is focused on performance measurement should be explicit about design issues. Otherwise the results of performance measurement can be misleading. Among the plethora of questions related to design issues, answers to the following four are key:

• *What are appropriate approaches to case mix adjustment?* This issue is crucial in adjusting for other influences in studies of the association between process measures and outcomes or in considering differences among client populations in comparisons across treatment facilities [40]. In addition to the usual client-level variables used in case mix adjustment (e.g., age, gender, co-morbidities), additional variables (e.g., client self-selection or level of substance use before entry into treatment) also may be necessary to properly adjust for case mix for substance use disorders. In particular, case-mix adjustment methods should be further developed that have the following characteristics: availability in commonly used datasets (currently this generally means administrative data), illuminate any relevant disparities in treatment (e.g., racial/ethnic, gender, age) and can be targeted to specific populations (e.g., adolescents, pregnant women, individuals who are homeless). At the same time, careful consideration needs to be given to the potential for case mix adjustment to mask important differences across groups.

• *What contributes to variation in performance measures?* This research is important for driving not only accountability but quality improvement efforts as well. Issues for consideration include how to attribute performance results when treatment for substance use disorders often is offered by a team of clinicians or how to interpret results when clinicians may see too few clients to make the statistics meaningful [41]. Beyond the immediate influence of clinician/client interactions, it also is key to understand the extent to which variations in performance measures results can be attributed to system-level, facility-level, community-level, and client-level factors and how these can be teased out.

• *How is research influenced by the timing of data collection during the course of treatment and follow-up*? By carefully considering timing of data collection, researchers can address questions such as what is the relationship between process measures and outcomes related to substance use during or shortly after treatment and more distal functional outcomes. However, constraints may be introduced by the availability of data. For research using secondary data sources, data may be collected only at admission, at 90-day follow-up periods, at time of treatment service, or on an outcome event basis (e.g., date of arrest).

• *How does the definition of a treatment episode influence the development of performance measures?* Decisions on when to collect outcome data or assess process measures need to be considered in the context of how episodes of treatment for substance use disorders are defined (e.g., including only one cycle of treatment or also including a period of recovery), what constitutes termination of treatment and how recovery is conceptualized [37,42,43].

#### Use of Electronic Health Records (EHRs) to incorporate new sources of information

New information for performance measurement is on the horizon with the accelerated pace of dissemination of EHRs, yet most current performance measures are focused on existing administrative data sources. EHRs have enormous potential to expand types of information, some of which is impossible to envision today including collection of more in-depth information, text mining of information from clinicians’ notes using natural language programming, blending of data across sites and systems of care (subject to confidentiality restrictions), and ongoing data collection during the course of treatment. This information, in turn, will allow for new measure development and implementation.

Thus, it is critical for those interested in research focused on performance measures for substance use disorders to: track what areas are being targeted for inclusion in EHRs; carefully assess what information should be collected and what is actually being captured; ensure the participation of providers of services for substance use disorders in EHRs; and monitor the pace of implementation. Recently, the NIDA Clinical Trials Network (CTN) undertook a consensus process to develop a treatment-relevant set of common core data elements with standardized vocabularies relevant to drug addiction treatment that could be incorporated and widely adopted into harmonized electronic medical record systems [44]. Incorporation of information related to substance use disorders into mainstream medical EHR systems, would open up the possibility for the new development and research on composite measures that aggregate a focus on substance use disorders and medical conditions (e.g., diabetes, hypertension, depression).

Despite the fast pace of EHR development and its promise to improve the quality or performance measurement, it is important to guard against inflated expectations. In the short term, the standardized performance measurement that is essential for accountability will still rely on more traditional information sources, e.g., claims/encounter datasets or patient surveys. At the same time, collection of expanded information and new approaches to collecting data are also important in order to exploit the new opportunities that EHRs offer. As EHRs become more prevalent, the answers to the following questions need to be considered:

• *What are treatment programs’ and clinicians’ perceptions of clinical decision-making software or performance measures that are fed back to them through EHRs?* Qualitative studies are needed immediately to explore treatment programs’ and clinicians’ views about how incorporating into EHRs performance measures or clinical decision-making software that is designed to improve performance can help their care of individuals with substance use disorders. These studies, conducted in environments both before and after the implementation of EHRs, can help to identify treatment program administrators’ and clinicians’ impressions of the capacity of EHRs; explore variations in these impressions across geographic areas to discern if there are local cultures around appropriate treatment; and evaluate if clinicians’ initial impressions are confirmed when EHRs actually are implemented.

• *How do clinicians and clients interact with EHR software that feeds back information to them on performance measure scores?* Research could offer input to the design of EHR features in terms of how clinicians and clients interact with the software, e.g., how do clinicians and clients engage with web-based tools for screening and reporting results of screening, treatment of substance use disorders, communication between clinicians and clients, or communication among clients or among consulting clinicians?

• *What is the validity of performance measurement data collected through EHRs?* Despite all the possibilities that are and will become available through EHRs, data validation still will be needed because the new technology is not a panacea [45]. Issues will remain such as clients’ accurately recalling or truthfully revealing their use of alcohol or drugs or clinicians responding to stigma by failing to record drug or alcohol abuse.

• *How might EHRs be designed and implemented to deal with 42CFR regulations*? For example, decision support software could be incorporated to prompt clinicians to get explicit approval for sharing data.

#### Connection with broader measurement efforts

The treatment of individuals with substance use disorders has never been limited to specialty substance abuse treatment settings. The impetus for integration with general medical care has accelerated, however, with the national support for screening and brief intervention in primary care settings [46], the development of health homes (also known as medical homes) that are accountable for individuals and their overall care, and a renewed focus on community health centers as sites of services [47]. Moreover, there is a renewed focus on care coordination with the publication of a framework for measurement and compendium of existing measures [48]. Researchers need to consider the following questions, working collaboratively with the addiction field, those focused on performance measures for medical conditions, and those focused on performance measures for care coordination:

• *What are ways that the recognition of substance use disorders influences performance measures in other areas within the health care and in other sectors* (e.g., success in employment/school)? Specifically, what is the impact of diagnosis of substance use disorders on medical care quality, especially for chronic medical conditions?

• *How is the quality of treatment for substance use disorders related to the quality of medical treatment including preventive services or treatment for chronic conditions?* For example, are organizations that provide better quality of treatment for substance use conditions also the ones providing better quality of treatment for other conditions? Does better quality of treatment for substance abuse conditions result in better outcomes for other conditions, such as diabetes or mental health conditions?

• *What are key transitions and linkages for which performance measures should be developed:* between levels of care in specialty treatment for substance use disorders; between sectors of the substance use and general medical treatment systems; and among other systems that impact clients (e.g., educational, criminal justice, and substance use prevention)?

• *How can data be integrated and coordinated, as a basis for performance measures across treatment settings* (e.g., medical or health homes, specialty medical settings, specialty behavioral health settings, and primary care) and across systems (e.g., treatment settings, criminal justice, and housing)?

### Implementation of performance measures for accountability and quality improvement

The third main area of recommendations for research is focused on what facilitates or impedes successful implementation of performance measures. For widespread adoption, implementation also warrants attention [2,49,50]. In this area, important lessons may come from best practices for implementation borrowed from general medicine, education, business/industry, manufacturing, or agriculture. Examples of best practices related to performance measures from other sectors may offer lessons on determining which incentives are most effective, setting goals, dealing with poor performance or feeding information back to treatment providers and clinicians.

In this section we first focus on descriptive studies that are needed to understand the current environment in which implementation of performance measures could take place. Next, we outline additional work related to implementation that pertains to capacity requirements, reporting on the results of performance measures to providers and the public, the role of incentives in improving performance, and the impact of public policies and cost considerations on the implementation of performance measurement.

#### Understanding the current implementation of performance measures

Despite widespread use of performance measures, there is not yet systematic understanding of where and how they have been implemented. This area of fast-paced change makes the design of these studies challenging in order that they not be obsolete as soon as they are completed. Studies focused on current implementation are needed to describe:

• *What performance measurement is currently underway, by which groups using which performance measures* (e.g., specialty treatment programs, Medicaid, commercial health plans, or states)?

• *What results of performance measurement are available and how are they used*.

• (e.g., public reporting, quality improvement efforts, incentive payment initiatives, or accountability for payment)?

• *What are various stakeholders’ roles in determining which performance measures to adopt and educating clinicians and treatment programs about the measures* (e.g., state agencies, providers, consumers)?

• *What level of effort is in place, particularly in state agencies, to implement performance measures in terms of staffing, data capacity, and evaluation of the implementation* (e.g., training and technical support related to the use of measures)?

#### Understanding capacity requirements for using performance measures

In order to implement performance measures, multiple players need a basic capacity to collect data, calculate measures and use these measures. Therefore, studies of current capacity of states, treatment providers and others also are required regarding:

• *What minimal or optimal infrastructure is needed to implement performance measurement? What is the current capacity of specialty treatment programs for performance measurement, and what capacity is needed including minimum size (numbers of clients served), technology, staffing and training in data collection and interpretation, or resources?*

• *What are states’ and treatment programs’ current capacities to interface with each other, especially as this relates to using newer technologies such as EHRs as a tool for performance measurement?*

• *How do the methods by which performance measures are selected (*e.g.*, imposed vs. chosen with clinician and treatment program participation) influence use of measures and improvement in performance?*

• *How do different levels of performance monitoring, reporting or accountability impact performance (*e.g.*, treatment program, clinician group, or specific clinician levels)?*

• *What is the impact on implementation of performance measures of organizational culture/leadership, clinician education, incentives for adoption or sanctions for lack of adoption of performance measures, or participation in national programs such as NIATx?*

#### Reporting results of performance measures

The impact of reporting to consumers, clinicians, hospitals and health plans has been widely studied in the medical sector. Reporting on the impact of performance measures for substance use disorders is much less prevalent, although some states make provider-level information available to the general public (e.g., North Carolina and Oklahoma) and client-specific information to providers only (e.g., Oklahoma) [14]. Currently, there is little research on these reports in terms of how often providers or the public access them and how they use them for quality improvement, accountability or selecting sites to seek treatment. Knowledge of consumers’ understanding and use of performance measures may be an important component of developing new approaches to patient centered care.

Moreover, it is well accepted that clients with substance use disorders differ from those seeking medical care in terms of readiness for treatment, co-occurrence of mental health problems, and coercion into treatment. How these differences influence consumers’ use of public reports on the performance of treatment programs or clinicians has not yet been studied. Thus, research addressed at answering the following questions would be fruitful both in terms of reporting to consumers and reporting to treatment programs and clinicians.

• *What information do consumers of services to treat substance use disorders, or their families, want or need when they look for treatment services?*

• *What would increase consumers or their families to awareness and use of publically reported information?*

• *What is the impact of providing feedback on performance to treatment programs in terms of changes in scores on process-focused performance measures or clients’ outcomes?*

• *How do we package data for treatment programs or clinicians to most likely be used for performance improvement efforts?*

• *How can EHRs be used to offer client-specific feedback regarding meeting performance measures requirements to clinicians on a timely basis to facilitate the use of the information for quality improvement?*

#### Incentives for performance improvement

Performance-based contracting, in which payment is contingent upon meeting specified performance levels, has been implemented for general medicine [51-53] and included in health reform legislation and several ongoing demonstrations at the federal level [54]. Effectiveness evidence is mixed, however [55].

The lag in pay-for-performance focused on substance use disorders has been due, in part, to less consensus on performance measures and improvement strategies [56] and less suitable state data to support it. Nonetheless, some states have implemented this approach: Delaware had adopted it for outpatient treatment programs but recently dropped it [57,58]; Maine implemented performance-based contracting for substance abuse in the early 1990’s and a restructured version in 2007 [59-61]; Connecticut had a pilot project on connection to a lower level of care after intensive residential treatment and readmission to detoxification; and Massachusetts is in the planning stages [62]. In spring 2011, SAMHSA announced new requirements in the Block Grant applications for States to collect performance and outcome data which ultimately will be used to determine if States receive an incentive based on performance [63].

Incentive-based payment approaches will be promulgated more widely, and thus research should address a broad range of questions listed below. Ideally, researchers could capitalize on new implementation of incentive-based payment schemes to embed randomized design to examine the impact on quality. The questions listed below are focused on the implementation of incentive-based payment systems as they relate to selecting performance measures and eliciting participation. Design of the incentives is beyond the scope of this paper.

• *What performance measures for substance use disorders currently are being used for incentive-based payments?*

• *Are incentives based on performance measures effective ways of promoting adoption of evidence-based practices and new technologies by clinicians who treat individuals with substance use disorders?*

• *What is the role of public recognition through reporting on performance measures in influencing performance improvement for facilities and clinicians?*

• *What are unintended consequences of incentivizing specific aspects of performance* (e.g., changes in performance in areas that are not the focus of the performance measure)?

• *What is the impact on client access and outcomes when linking funding to different types of performance measures* (i.e., structure, process, or outcome)?

• *What elements in designing the implementation of incentive-based schemes that might promote sustainability?* (e.g., involving end users in selecting and specifying the performance measures).

• *How might incentives be designed to focus on performance measures based on proximal (during treatment) outcomes rather than longer term outcomes?*

#### Policy and cost impacts on implementation of performance measures

Policies at the federal, state and local levels, in addition to the cost of performance measurement, potentially can influence their implementation. Research understanding these influences is necessary for any successful implementation of measures. Potential research questions include:

• *How do federal, state and local policies drive the types of performance measures for substance use disorders that are implemented?* As noted above, SAMHSA’s changes to the Block Grant applications for both substance abuse and mental health (which optionally can be combined), will place a greater emphasis on performance measures at the state level.

• *What is the impact of health reform legislation*? With the new emphasis under health reform on health homes and on integration of primary and specialty care, research is needed to evaluate how performance measures are used in these settings.

• *Do structural barriers on treatment retention bias possible improvement in performance measures because individuals may not receive treatment for services not covered by their insurance policies* (e.g., treatment for relapse management)?

• *What are the cost implications of the inclusion of performance measures for treatment programs* (e.g., data collection, staff time, interpreting and acting on results)?

• *Does the cost of implementing performance measures at the state or treatment provider level outweigh the potential improvements in quality of client treatment and savings through more efficient provision of services?*

#### Barriers and facilitators to measure development and research

The complex health care environment presents challenges. Thus, the meeting participants also focused on the need for understanding the barriers and facilitators for research to support measure development and implementation. Investigation would be useful in studying the following areas:

• *What steps are key in developing and implementing performance measures for substance use disorders so that the time period from measure conceptualization, specification, testing and implementation can become more rapid?*

• *What are specific audiences’ needs and the implications for measure development and implementation that is more audience targeted?* For example, might practitioners and consumers become more involved in driving the development of measures, e.g., by exploring some of the basic tenants of participatory action research so that the users of measures are involved at the inception of measure development? Or, might treatment program directors be included in development so that the information they need to make crucial decisions is included? Or, might clinicians weigh in on the issue of considering clients’ outcomes throughout the treatment process?

•*What are the issues to consider in developing performance measures for substance use disorders so that the impact of unintended consequences can be minimized?* (e.g., measures that are not as easily “gameable” through careful specification and testing).

• *What is the landscape on obstacles to information exchange or data sharing that may preclude performance measurement and/or protect clients,* particularly barriers to sharing information outside of specific provider settings, between specialty treatment and general medical treatment settings, and across sectors such as integrating data from educational systems? Both practical issues with data sharing and legal issues such as 42 CFR structures need to be better understood [64].

• *With a new focus on EHR, might treatment programs or clinicians be prompted to elicit clients’ permission to share clinical information?*

## Conclusion

Research focused on performance measures for substance use disorders is both exciting and challenging because it is conducted in a context that is changing in some ways that we can anticipate and other ways that we cannot easily envision. Performance measurement for substance use disorders will need to adapt to new approaches to treatment, federal regulations on parity for behavioral health care, national health reform, and an atmosphere of heightened interest in quality and performance measures across the health care system. Given the National Quality Strategy [65] and SAMHSA’s response [66], performance measures need to place substance abuse treatment in the context of person-centered care, which implies active roles for individual clients and their families.

New measures will need to be developed and current measures will need to be refined to take into account new treatment approaches, such as the use of electronic communication between providers and clients using new technologies such as tablet computers and smart phones that were not available even a few years ago. However, dissemination of these innovations is not universal. For example, electronic health records hold promise for breakthroughs in performance measurement and quality improvement, but they are not yet widely used or available within specialty substance use treatment.

Often tradeoffs will need to be considered between the simplicity of a performance measure that may be easier to implement more broadly versus a more complex measure that may be better able to capture clinical quality but that may be more difficult to implement. Therefore, research needs to be grounded in the current environment and also forward looking in order to be useful in a rapidly changing healthcare system.

Within this complex and changing environment, the meeting reported on here offers a snapshot of current topics and suggestions for research questions. We hope that if these areas are studied, performance measures for unhealthy substance use and substance use disorders will better serve to support our common goals -- improving access and quality of care for individuals with substance use disorders.

## Competing interests

The authors declare that they have no competing interests.

## Authors’ contributions

DWG chaired the committee that planned the meeting, chaired sessions during the meeting and drafted the manuscript. CMH participated in meeting planning, presented background materials, led breakout groups, and drafted the manuscript. AA participated in meeting planning, prepared background materials, compiled notes from the meeting, and participated in drafting the manuscript. FM participated in meeting planning, served as co-chair, led breakout groups and participated in drafting the manuscript. CW participated in meeting planning and in drafting the manuscript. All authors read and approved the final manuscript.
